# Re‐establishing the pecking order: Niche models reliably predict suitable habitats for the reintroduction of red‐billed oxpeckers

**DOI:** 10.1002/ece3.2787

**Published:** 2017-02-23

**Authors:** Riddhika Kalle, Leigh Combrink, Tharmalingam Ramesh, Colleen T. Downs

**Affiliations:** ^1^ School of Life SciencesUniversity of KwaZulu-NatalScottsvillePietermaritzburgKwaZulu-NatalSouth Africa; ^2^ School of Ecology and Environment StudiesNalanda UniversityRajgirBiharIndia; ^3^ The Endangered Wildlife TrustModderfonteinSouth Africa

**Keywords:** conservation, lethal agrochemicals, obligatory mutualist, oxpecker, reintroduction success, species distribution models

## Abstract

Distributions of avian mutualists are affected by changes in biotic interactions and environmental conditions driven directly/indirectly by human actions. The range contraction of red‐billed oxpeckers (*Buphagus erythrorhynchus*) in South Africa is partly a result of the widespread use of acaracides (i.e., mainly cattle dips), toxic to both ticks and oxpeckers. We predicted the habitat suitability of red‐billed oxpeckers in South Africa using ensemble models to assist the ongoing reintroduction efforts and to identify new reintroduction sites for population recovery. The distribution of red‐billed oxpeckers was influenced by moderate to high tree cover, woodland habitats, and starling density (a proxy for cavity‐nesting birds) with regard to nest‐site characteristics. Consumable resources (host and tick density), bioclimate, surface water body density, and proximity to protected areas were other influential predictors. Our models estimated 42,576.88–98,506.98 km^2^ of highly suitable habitat (0.5–1) covering the majority of Limpopo, Mpumalanga, North West, a substantial portion of northern KwaZulu‐Natal (KZN) and the Gauteng Province. Niche models reliably predicted suitable habitat in 40%–61% of the reintroduction sites where breeding is currently successful. Ensemble, boosted regression trees and generalized additive models predicted few suitable areas in the Eastern Cape and south of KZN that are part of the historic range. A few southern areas in the Northern Cape, outside the historic range, also had suitable sites predicted. Our models are a promising decision support tool for guiding reintroduction programs at macroscales. Apart from active reintroductions, conservation programs should encourage farmers and/or landowners to use oxpecker‐compatible agrochemicals and set up adequate nest boxes to facilitate the population recovery of the red‐billed oxpecker, particularly in human‐modified landscapes. To ensure long‐term conservation success, we suggest that the effect of anthropogenic threats on habitat distributions should be investigated prior to embarking on a reintroduction program, as the habitat in the historical range may no longer be viable for current bird populations.

## 1 Introduction

Globally, anthropogenic rather than climate‐mediated habitat modification appears to be driving range contraction in many bird species (Newbold et al., 2014; Okes, Hockey, & Cumming, 2008). Habitat suitability modeling is being actively applied to conservation planning and reintroduction programs to recover populations of species dwindling with contracted ranges (Cook, Morgan, & Marshall, 2010; Olsson & Rogers, 2009; Osborne & Seddon, 2012). Although reintroductions aid in expanding a species’ range, it is essential that the receiving areas are suitable to ensure that the new founder population will establish and thrive in the long term, with minimum interventions in the future (Armstrong & Seddon, 2008; Osborne & Seddon, 2012; Robert et al., 2015; Weeks et al., 2011). With changing climate and habitat conditions, the extent of species occurrence, resilience, persistence, and dispersal to establish new populations at new sites, postreintroduction, are vital measures of reintroduction success (Cade & Burnham, 2003; Sánchez‐Lafuente, Valera, Godino, & Muela, 2001; Weeks et al., 2011). Thus, failure to distinguish suitable and unsuitable habitat for self‐sustainability of the reintroduced population could eventually hamper the species’ conservation success (Robert et al., 2015; Soorae, 2013). Site unsuitability and anthropogenic pressure, impacting the fine‐scale habitat use of reintroduced birds, were some of the major reasons for the partial success or failure of bird reintroduction projects (Soorae, 2013). In order to address future bird reintroduction projects, we investigated the reintroduction success of the red‐billed oxpecker (*Buphagus erythrorhynchus*) in South Africa, as a case study for our habitat suitability models.

**Figure 1:**
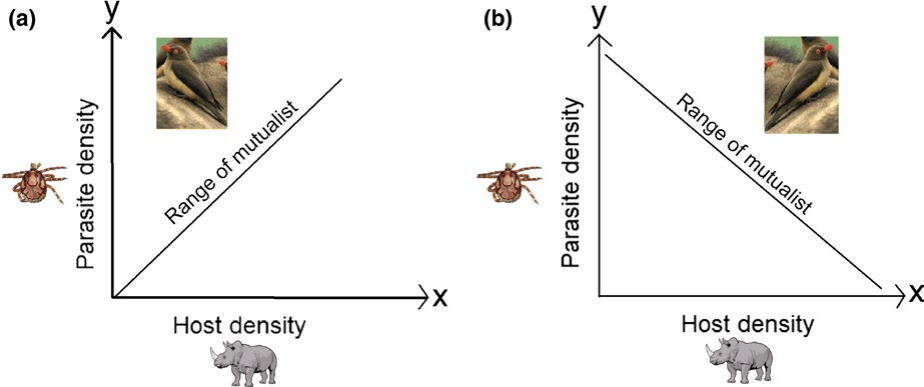
Theoretical expectations about the underlying mechanisms of range changes in obligatory mutualists. (a) Typically host and tick densities are directly proportional positively where we expect red‐billed oxpecker (RBO) to occupy their full range, (b) with human interventions, in this case, the excessive use of oxpecker‐incompatible agrochemicals, we expected a negative relationship, where the decrease in tick densities results in population decline and consequently range contractions of the RBO

The red‐billed oxpecker (hereafter called RBO and/or RBOs) and yellow‐billed oxpecker (*B. africanus*) are the world's only obligate mammal gleaners, endemic to the African continent (Dean & MacDonald, 1981). As keystone obligatory mutualists, they have symbiotic relationships with mammalian hosts by emitting antipredator warning calls and feeding on hard ticks to reduce the tick load (Bezuidenhout & Stutterheim, 1980). Many wild vertebrate host species are endangered, and as part of conservation efforts for threatened large mammals, a common practice is the removal of ectoparasites through chemical treatment, with devastating impact on hard‐tick populations (Ixodides, *Hyalomma* spp, and *Amblyomma* spp) (Mihalca, Gherman, & Cozma, 2011). However, this practice can lead to the population decline of avian consumers of ectoparasites (Bezuidenhout & Stutterheim, 1980) such as the RBO, which has suffered a significant population decline throughout most of its global range and in South Africa (Feare & Craig, 1998), primarily as a result of the elimination of many wild host species (large game, such as the white rhinoceros *Ceratotherium simum*) and from the indiscriminate dipping of cattle with products that were toxic to the birds. The major factor influencing the decline of RBOs was the increasing use of acaracides (mainly arsenical compounds) in cattle dips from 1902 onward (Bezuidenhout & Stutterheim, 1980). With the advent of new dipping compounds, lethal to ticks, but not the birds, the concept of re‐establishing RBOs through reintroduction efforts within their historic range became a viable option for increasing their distribution. Currently, dips containing organophosphates, organochlorines, or home brews (where farmers mix chemicals to make their own homemade dips) are the main threat to populations of RBOs in certain parts of South Africa. It is expected that the decline in host–parasite densities due to the aforementioned threats has caused range contractions of the RBO (Figure 1).

The Endangered Wildlife Trust (EWT) has been capturing and releasing RBO from source populations (i.e., areas of abundance) to areas within their historical range (The Endangered Wildlife Trust, 2010) from which they have been eliminated by inappropriate livestock dipping practices and oxpecker‐incompatible pesticide use (Bezuidenhout & Stutterheim, 1980) since 2002. The receiving areas are sensitized by farmer awareness programs as a means to ensure that only products considered oxpecker‐compatible will be used in the areas where the birds are released. Quantitative information on the ecological niche of the species across vast geographic space could help restore locally extinct populations and prioritize regions for efficient management. In order to effectively direct conservation efforts for RBOs in South Africa, it is important to make realistic predictions in our niche models. Thus, suitable habitat of RBOs should include abiotic data (climate, topography, and habitat) comprising of high‐resolution remote‐sensing data, biotic consumable resources (i.e., ticks as prey), and other biotic interactions (i.e., co‐occurring species and host distributions). With the development of ecological theory and advanced niche modeling approaches, the role of interaction strength within mutualistic species (such as biotic predictors) and biophysical interactions must be reflected in distribution models for mutualists in order for one to make realistic predictions at large spatial scales, needed for sound conservation planning. We applied predictive distribution modeling techniques; generalized linear models (GLM), generalized additive models (GAM), boosted regression trees (BRT), and ensemble models using RBO occurrence data to develop habitat suitability maps taking important biotic–abiotic variables into account, to aid the ongoing reintroduction program, and to develop recovery strategies for the RBO in South Africa. We also tested the model's ability to reliably predict suitable habitat in reintroduction sites. We predicted that biotic–abiotic variables were influential in the distribution of RBOs. We predicted that suitable habitat of RBOs would include nest‐site characteristics (high tree cover, savanna, and woodland habitat), bioclimate (high temperature and rainfall), consumable resources (host–tick density), and proximity to protected areas.

## 2 Materials and Methods

### 2.1 Presence/absence records of RBO

The sources of occurrence records included field data and citizen science data. Field data included the ongoing reintroduction programs (i.e., capture and release/translocation records) by the EWT (2007–2014) and bird ringing operation data (2007–2014) housed by the South African Bird Ringing Unit (SAFRING). Other presence records were obtained from citizen science data (2007–2014) of the South African Bird Atlas Project (SABAP2, http://sabap2.adu.org.za/). The absence records were taken from SABAP2. Presence and absence records are detections and nondetections from the citizen science survey and except for a few records of known absences, we cannot be certain that these absence records represent true absences. All the records from the aforementioned sources were pooled together and plotted using ArcMap 10 (ESRI, 2012). This gave us 1295 records of RBO presence for our modeling (Appendix S1).

### 2.2 Biotic and abiotic predictors

A systematic literature search based on ISI web of knowledge using the keywords “red‐billed oxpecker and ecology” and “red‐billed oxpecker” aided in gathering information on ecological predictors. We synthesized and related relevant life‐history information on RBOs from the available literature, as a basis for designing and interpreting the habitat models. Variables either known or suspected to correlate with RBO occurrence were considered (Appendix S2). Some of the key requirements for successful reintroductions of RBOs would be a fairly high density of wild or domestic host species (Nunn, Ezenwa, Arnold, & Koenig, 2011; Plantan, Howitt, Kotzé, & Gaines, 2014; Stutterheim, 1981; Weeks, 1999), adequate tick densities as food (Bezuidenhout & Stutterheim, 1980), suitable nest sites (Stutterheim, 1982), suitable land uses, open savanna habitat (Sirami & Monadjem, 2012), protected areas (Stutterheim & Stutterheim, 1980), and water sources (Stutterheim, 1981). We compiled recent occurrence records (2007–2014) on 20 symbiotic mammal species and six tick species from various sources (see Appendix S2 for details). Occurrence records on mammal symbionts/wild host species were obtained from Ezemvelo KwaZulu‐Natal Wildlife, Mammal Map group (University of Cape Town), and the Durban Natural Science Museum. All the host species records were pooled together, and host density was calculated as a spatial layer using the “Kernel density” tool of the Spatial Analyst extension in ArcMap 10 (ESRI, 2012), giving us a smooth surface raster, as an index of abundance. *Amblyomma hebraeum* is the most numerous tick species on cattle, and along with *Rhipicephalus* (*Boophilus*) *microplus* and *B*. *decoloratus* are abundant on wild host species, mainly large mammals such as giraffe (*Giraffa camelopardalis*), eland (*Tragelaphus oryx*), bushbuck (*T. scriptus*), African buffalo (*Syncerus caffer*), warthog (*Phacochoerus aethiopicus*), Burchell's zebra (*Equus burchelli*), nyala (*T*. *angasii*), and kudu (*T. strepsiceros*) (Horak, Macivor, Petney, & Devos, 1987). *Rhipicephalus* (*B.) microplus* feeds more efficiently on cattle (Aguirre, Gaido, Vinabal, De Echaide, & Guglielmone, 1994). The brown ear‐tick (*R. appendiculatus*) and red‐legged tick (*R. evertsi evertsi)* feed over giraffe (*G. camelopardalis*), bushbuck (*T. scriptus*), kudu (*T. strepsiceros*), African buffalo (*Syncerus caffer*), nyala (*T*. *angasii*), and eland (*T. oryx*) (Horak, Golezardy, & Uys, 2007; Horak, Potgieter, Walker, De Vos, & Boomker, 1983). Bont tick (*A. hebraeum*), blue tick (*B. decoloratus*), brown ear‐tick (*R. appendiculatus*), and red‐legged tick (*R. evertsi evertsi*) are preferred by RBO (Bezuidenhout & Stutterheim, 1980; Plantan, 2009; Stutterheim, Bezuidenhout, & Elliott, 1988). We calculated tick density following the same approach used to calculate host density. Presence/absence records of six species of starlings were obtained from SABAP2 from 2007 to 2014. We considered six widely distributed starling species as a proxy for suitable nesting sites because RBOs belong to the starling family, and they are secondary cavity nesters. Presence/absence records of starling species were pooled together to calculate the starling density following the same procedure used to calculate host and tick density.

In addition, we used 19 bioclimatic variables from WorldClim 1.4 (Hijmans, Cameron, Parra, Jones, & Jarvis, 2005). Digital elevation data at 90‐m resolution were used to quantify mean elevation, with elevation and aspect being the topographical variables (see Appendix S2 for details). RBOs follow the movement of their mammal symbionts when surface water availability fluctuates seasonally, causing a shift in local movements and when water supply decreases. RBOs frequently visit large rivers, often where large game congregate (Stutterheim, 1981). Euclidean distance to rivers and protected areas was calculated using the “Euclidean distance tool” to create a raster “distance to” (km) layer, respectively. Surface water body records were obtained from a national database which was then used to calculate the surface water body density (see Appendix S2 for details). Surface water body density and distance to river measurements were considered as variables of water sources. Vegetation variables included land cover, biomes, and tree cover (see Appendix S2 for more details). All explanatory variables were clipped to South Africa. Individual raster layers were created for each variable using the Zonal Statistics tool in Spatial Analyst, ArcMap 10 (ESRI, 2012). Multicollinearity between predictors can be problematic for parameter estimation, as it inflates the variance of regression parameters and leads to misidentification of relevant predictors in a model (Dormann et al., 2013). To avoid problems of multicollinearity in our models, we used the ellipse‐shaped glyphs and Pearson correlation coefficients using the package “ellipse” (Murdoch & Chow, 2007) to remove variables with a correlation ≥.7 (Appendix S3). The spatial autocorrelation of RBO presence records was assessed with the Moran's *I* statistic in ArcMap 10 (ESRI, 2012).

### 2.3 Species distribution modeling

The relationship between abiotic–biotic variables and presence/absence of RBOs was analyzed using various techniques. Although there is spatiotemporal mismatch in our occurrence records as well as our predictor data sets as they were collected from multiple sources representing different methodologies and sampling effort, we assumed that all these records were constant across space and time. We included various combinations of predictors selected randomly in our models. To avoid overfitting in parametric models, we reduced complexity in GLM (McCullagh & Nelder, 1989) and GAM (Wood & Augustin, 2002) models by serially removing variables from a full model until a minimum Akaike information criterion (AIC) was achieved. In GAM, we used the automatic term selection procedure that enforces a penalty to smooth functions and efficiently eliminates terms from the model (Wood & Augustin, 2002). In GAM, the dimension of the basis used to represent the smooth term (*k*) was set to 5. Models were constructed in R version 3.11 (R Core Development Team, 2013) with packages “mgcv” (Wood, 2011), “gbm” (Ridgeway, 2013), and “dismo” (Hijmans, Phillips, Leathwick, & Elith, 2011). The “MuMIn” package (Barton, 2012) was used for model selection in GLM and GAM, providing AIC_c_ values (corrected for small sample sizes) and a ranked selection table for all possible combinations of variables (i.e., candidate models). Candidate models with ΔAIC_c_ ≤2 were considered the best models (Burnham, Anderson, & Huyvaert, 2011). We applied the effect function from the “effects” package in R (Fox, Weisberg, Friendly, & Hong, 2014) to the best‐supported GLMs. We used the functions “allEffects” and “plot” to the constructed top model objects to display the graphic effects of any relationships between predicted probabilities, predictor sets, standard errors, and confidence intervals.

Boosted regression trees models were run following the scripts in Elith, Leathwick, and Hastie (2008). Models were again constructed in R using packages, “gbm” (Ridgeway, 2013), and “dismo” (Hijmans et al., 2011). To increase the interpretability of the models, predictor sets were reduced using the “gbm.simplify” function (Elith et al., 2008). Using the reduced sets of variables, we fitted BRT models with a learning rate of 0.005, a tree complexity of 5 (the number of splits in each tree, also called the interaction depth), and a bag fraction of 0.5 (the default setting of the fraction of the training set observations randomly selected to propose the next tree in the extension), as suggested by Elith et al. (2008). We included land cover and biome as factor variables in all our models. We performed cross‐validation optimization using a family of Bernoulli. This created 10 initial models of 50 trees. All other parameters were left at default settings. The final model was fitted with 3,600 trees. We applied BRT models to explore important interactions of predictors. We calculated the relative variable importance using the function “varImpBiomod” (Thuiller, Lafourcade, Engler, & Araujo, 2009). The relative importance (%) of each variable in the best model was normalized to 100, with higher numbers indicating stronger influence on the response variable. We used the partial response plots to visualize the relative importance of the predictors to interpret the fitted functions in BRT and GAM.

We used an “ensemble” approach (Araújo & New, 2007) to combine predictions from multiple top performing models that varied in structure and parameterization, as this is often more robust than predictions from a single model. Ensemble predictions were calculated with weights assigned to each modeling technique based on its discriminatory power, as measured by the area under the receiver‐operated characteristic curve (Araújo & New, 2007). The data set was randomly divided into training (75%) and test set for model evaluation (25%). We looked for agreement and disagreement among models to reliably predict suitability at the reintroduction sites.

### 2.4 Model evaluation and calibration

We assessed the model performance based on the accuracy of predictions for both the training and the independent test data and reported the area under the receiver‐operating characteristic curve (AUC) as discrimination performance criteria. AUC values range from 0 to 1, where the value of 0.5 indicates that a model performs the same as a random assignment and that values above 0.5 indicate increasing model discrimination between presences and absences; values below 0.5 indicate a reversed favoring of observations, with presences receiving lower fitted values than absences. AUC scores have been widely used in comparing species distribution models, but have been criticized (Allouche, Tsoar, & Kadmon, 2006). We assessed model discrimination based on how well the models accurately predicted the training and test data set according to the value of kappa. The kappa values range from −1 to +1, where +1 designates perfect agreement and values of zero or less designate a performance no better than random (Cohen, 1960). We reported kappa because it corrects for prediction success by chance and is considered a robust index in contrast to AUC (Manel & Williams, 2001). We calculated the Youden index, called the true skill statistic, as criteria for selecting the optimal cutoff value (i.e., the optimal threshold criteria called “Max sens + spec” as in Freeman and Moison (2008). This index identifies the threshold that maximizes the sum of sensitivity and specificity and thereby enhances the possibility to differentiate between presence and absence of a condition when equal weight is given to sensitivity and specificity. All model evaluation statistics and calibration plots were calculated and developed using the R package “PresenceAbsence” (Freeman & Moison, 2008).

## 3 Results

### 3.1 Variable importance and response curves

**Figure 2:**
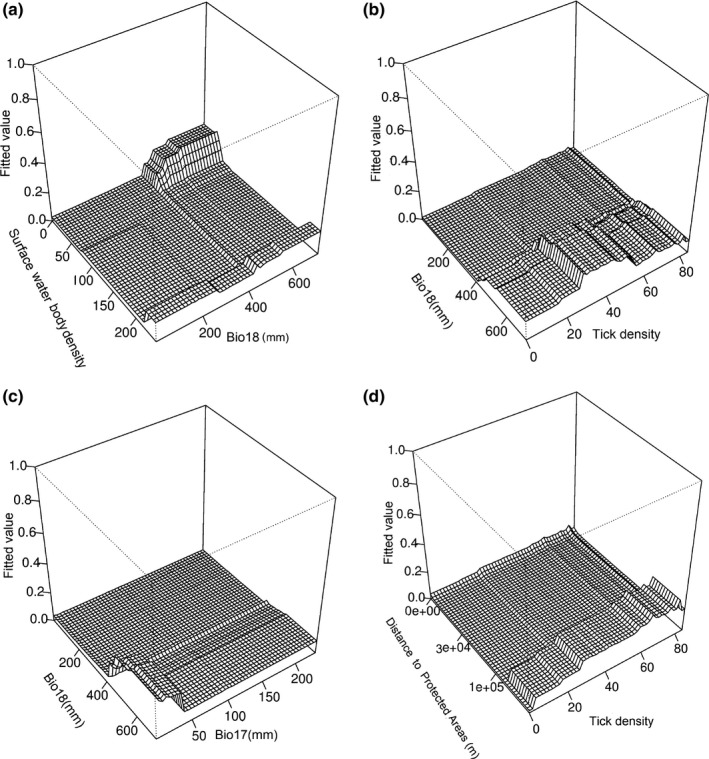
3D plots depicting interaction strength between influential predictors from the boosted regression trees (BRT) model. For explanations on abbreviations, please refer to Appendix S2

Spatial autocorrelation in RBO presence records was moderate (Moran's *I *=* *0.4), although significant. Abiotic–biotic variables were present in the top ranked model in GAM (*w* = 0.79) and GLM ([*w* = 0.76]; Appendix S4). Across all modeling methods, the relative importance of abiotic and biotic variables varied (Table S1 in Appendix S5), although overall tree cover, temperature, precipitation, biome, starling density, host density, tick density, proximity to protected areas, and land cover had significant contributions (≥5%) in our models. Woodland, thicket, savanna biome, and water bodies were predicted suitable habitats. Host density had a positive relationship with predicted occurrence of RBOs (Figures S1–S3 in Appendix S5). Predicted suitability increased with tree cover and then stabilized at 40% (Figure S3 in Appendix S5). Temperature seasonality (bio8 = 20–25°C) and areas receiving high summer rainfall (bio18 = 500–700 mm) were predicted suitable. Bio9 showed a bimodal response in GAM, while the predicted suitability was skewed (10–15°C) in BRT, and in GLM, it showed a positive relationship with predicted occurrence of RBOs. Bio7 showed a hump‐shaped curve in GAM, while it had a bimodal pattern in BRT. Bio17 had a negative relationship with predicted suitability. In BRT, predicted suitability was skewed to low precipitation at the coldest quarter (bio19 = <50 mm). Tick density had hump‐shaped responses in GAM and BRT, while having a weak relationship with predicted occurrence in GLM. Starling density had a hump‐shaped curve in GAM and BRT, while in GLM, the relationship was positive with predicted occurrence of RBOs. Suitable sites were identified both close to and away from protected areas, while GLM fitted a negative relationship with predicted occurrence. Surface water body density had a negative relationship in GAM and GLM, while in BRT, the high probability of RBO occurrence was skewed to high water body density. An elevation range from 1,500 to 2,500 m was predicted suitable for RBOs in GAM. There was a strong interaction strength between bio18 and surface water body density (interaction size = 123.12, Figure 2a), between bio18 and tick density (interaction size = 59.05, Figure 2) and between bio18 and bio17 (interaction size = 45.62, Figure 2c). The interaction strength between proximity to protected areas and tick density (interaction size = 31.26, Figure 2d) was moderate.

### 3.2 Model validation and extent of suitable habitat

High AUC values (>0.9) for all four models indicated that occupied sites were highly likely to be assigned a higher probability of presence than background sites irrespective of method. The calibration plots indicated that each of the tested models for RBOs performed well (Figure S4 in Appendix S6). All models had good accuracy (κ ≥ .5) with the test data. Cutoff values at .5 resulted in 41%–59% of the test data being correctly classified (Table S2 in Appendix S6).

**Figure 3:**
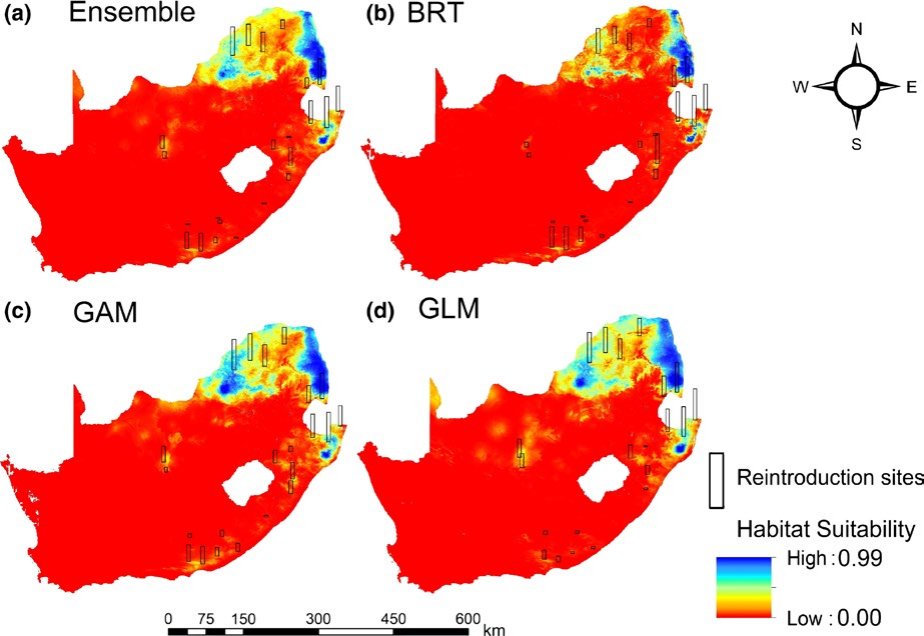
Habitat suitability map from (a) ensemble models, (b) boosted regression trees (BRT), (c) GAM, and (d) GLM for predicting the distribution of red‐billed oxpeckers (RBOs) in South Africa. The rectangular bars are the quantitative representation of the predicted suitability values from the particular model at each reintroduction site. The taller the bar, the higher the predicted suitability value

The present IUCN range of RBOs in South Africa (BirdLife International, 2012) encompasses 222,885.15 km^2^ (extent of occurrence) covering the Gauteng (1,313.07 km^2^), KwaZulu‐Natal (KZN) (36,515.46 km^2^), Limpopo (1,23,312.87 km^2^), Mpumalanga (34,963.97 km^2^), North West (26,156.27 km^2^), Northern Cape (619.37 km^2^), and Free State (3.07 km^2^) provinces. However, our predictions estimated the total suitable area (0.5–1) ranging from 42,576.88 to 98,506.98 km^2^ across all methods (Figure 3a–d) covering the majority of Limpopo, Mpumalanga, North West, a substantial portion of northern KZN and Gauteng. Ensemble, BRT, and GAM models predicted suitable areas in the Eastern Cape Province and southern areas in the KZN, that cover parts of the historic range. A few southern areas in the Northern Cape, that fall outside the historic range, had suitable sites for RBOs.

### 3.3 Ensemble models and model agreement

Differences in models were apparent, although this was primarily in the southern portion of South Africa that had fewer detections of RBOs. Predictions from the best niche models nearly appeared to match the “real world scenario” by adequately predicting suitable and unsuitable RBO habitat when locations of reintroduction sites (with a 25‐km buffer radius, according to the circular home range of RBO (Stutterheim, 1981), included the predicted suitable values from each model. Ten of 23 reintroduction sites (40%) were accurately predicted as moderate to high suitable areas (i.e., 0.4–1) in ensemble models, 12 of 23 (52%) in BRT, 14 of 23 (61%) in GAM, and 11 of 23 (43%) in GLM (Figure 3a–d). In the Eastern Cape, BRT and GAM predicted few suitable sites covering 96.32 km^2^ and 5 km^2^, respectively, while predictions for southern KZN spanned 51.6 km^2^ and 38.7 km^2^, respectively. GLM and ensemble models predicted low suitability areas in the Eastern Cape and KZN provinces. This suggests that BRT and GAM had higher predictive power than GLM and ensemble models for sites in the historic range, which have the potential for future reintroductions.

## 4 Discussion

The distribution of RBOs was best explained by a combination of environmental and biotic variables, which agreed with our predictions. The comparatively more liberal models, like BRT and GAM, aided in identifying potential new release sites for future reintroductions. Our correlates of recent RBO distribution showed that the national range of RBOs still remains contracted in comparison with its historic range, despite several long‐term efforts invested in reintroductions since 1988. The relative variable importance in relation to ecological requirements of RBOs aids in understanding model outputs, and in determining whether the predictors chosen have biological significance. Most of the suitable sites predicted by our niche models were in close proximity and away from conservation areas, provincial nature reserves, game reserves, and national parks, that are largely concentrated in these northern provinces, which indicates that RBO populations have fairly recovered in the northern provinces of South Africa. These areas have adequate open savannas, woodlands, tree cover, and host–tick densities, and these relationships agreed with our predictions. In Africa, ticks require moist conditions for survival and reproduction (Londt & Whitehead, 1972), which explains the interaction strength between tick density and water body density, as well as summer rainfall. Therefore, seasonality and climatic conditions are significant factors that could impact tick developmental stages and have an indirect effect on oxpecker foraging behavior, as they feed on both larvae and adult ticks.

Red‐billed oxpeckes were recorded beyond the range as suggested by the IUCN for South Africa. These records came from SABAP2 and the ongoing reintroduction efforts by the EWT. Assisted colonization is termed as the translocation of a species outside their native/historic range to protect them from various threats, such as climate change and human‐induced habitat change (Ricciardi & Simberloff, 2009; Seddon, 2010). RBO records in the Northern Cape, close to the national boundary, suggest that populations in Botswana are spreading toward South Africa, while the records in southern areas of Northern Cape are a consequence of the spread of an introduced population released at the Rooiport Game Reserve in Kimberley and at Mokala National Park. Assisted colonization of RBOs in the southern parts of the Northern Cape began in 2007 and these birds continue to persist in large numbers, suggesting that assisted colonization could help in the population recovery of species outside its historical range (Chauvenet, Ewen, Armstrong, Blackburn, & Pettorelli, 2013). This is especially true when sites in the native range are unsuitable, although it is essential that areas used for assisted colonization are monitored regularly (Chauvenet et al., 2013). The predicted suitability of RBOs in the Northern Cape, outside of the historic range, is linked to the increasing artificial woodlands (i.e., *Prosopsis* spp) with the spreading human habitats in the west of South Africa (Hockey, Sirami, Ridley, Midgley, & Babiker, 2011). This northward shift has also been reported in many other bushveld species in South Africa (Hockey et al., 2011).

In parts of the Eastern Cape Province, that were once historically suitable, the breeding success and abundance of RBO have been particularly low in comparison with the northern provinces, which nearly matched our expectations from the suitability maps. The use of oxpecker‐incompatible agrochemicals and heavy land transformation, through subsistence and commercial agriculture, are both major threats to RBOs throughout their range (Okes et al., 2008). Our maps showed that small game reserves and farmlands were also suitable reintroduction sites in landscapes where savanna/open woodland habitat has been lost. Much of the landscape has transformed in Eastern Cape and KZN, where sufficient tree cover is scarce, as most of the savanna habitat has been converted to croplands to boost agricultural development. There is a possibility that the proliferation of game farming in Eastern Cape and KZN could facilitate the expansion of RBO distributions, as they are an effective biological control method for ticks and readily switch from wild hosts to domestic hosts. In farmlands, strategic placement of nest boxes eases the pressure on competition for tree cavities suitable for nesting and facilitates breeding by RBOs, as artificial nest cavities are readily accepted by the birds (The Endangered Wildlife Trust, 2016). This is a prerequisite for receiving sites in the ongoing reintroductions by the EWT and this strategy would help restore populations in the Eastern Cape and southern KZN. It is possible that RBOs can co‐occur with other starling species, as their niches are not identical in terms of foraging habits and habitat preferences and this minimizes the chance of competition for space and resources. Species with dissimilar niches can coexist (Krebs, 2009), although Morelli and Tryjanowski (2015) show that this functions best at a local scale; therefore, it was not surprising to find a significant contribution of starling density in our predictions. Our models did not predict suitable areas in the Free State Province, and many areas inland, which were part of the historic range of RBOs, primarily due to the low tree cover, as a result of habitat conversion for farming. The ongoing reintroductions within their historic range, which include farmlands, facilitate the formation of new biotic interactions (RBO–domestic host interactions). This could impose positive or negative effects as there are regional differences in the attitudes of farmers toward RBOs. All these factors suggest that the wide variation in the climatic suitability, distribution of host–tick density, and threat levels between the northern provinces (Limpopo, North West, Northern Cape, Mpumalanga, Gauteng, and north of KZN) and southern provinces (southern KZN and Eastern Cape) will have implications on the conservation of RBOs.

Our conclusions on habitat suitability of RBO from ecologically relevant models constructed from multiple data sources include certain caveats. Overall, we have selected important variables useful for suitability mapping of RBO which although predicted at coarse resolution may have differential influence at fine spatial scales, one of the issues being that a large percentage of RBO records coming from citizen science data were collected at large spatial scales and not point level information such as those from SAFRING and EWT. Hence, it was essential to include EWT and SAFRING data in our analysis as SABAP2 records in Eastern Cape and KZN did not have adequate records of RBOs for making predictions and to also remove false‐negative records from SABAP2 data. Moreover, it was important to expand the species distribution through the identification of suitable habitat in these two provinces. Some of the variables such as density of host, ticks, and starlings are point densities and not empirically estimated population densities. On the other hand, proximity to protected areas and rivers and their influence on RBO occurrence was almost at the scale twice the size of its home range. Moreover, bioclimatic variables at large spatial scales cannot account for microclimate influence due to variation in local habitat and topography. These caveats put together could have impacted the accuracy of our predicted suitability maps. Yet, given the macroecological scale of our predictions, it is often not possible to have access to perfect data sets; however, we have tried our best to minimize the spatiotemporal discrepancies between data sets by matching the duration (2007–2014) of RBO occurrence records and our variables used in the suitability modeling.

### 4.1 Conservation implications

Our models that explicitly incorporated biotic relationships (trophic relationships and host affiliations), and biotic interactions with environmental factors, aided in creating realistic species distribution patterns for RBOs. Models had good predictions of suitability at reintroduction sites on a national scale. This paves new directions for conservation efforts that make use of niche models with high predictive power. Our countrywide approach to suitability mapping helps delineate recommendations for future reintroduction plans, aimed at spatially integrating the major components of the target species’ distribution. Our maps clearly showed the northern provinces as hot spots of population recovery, which would not have been possible if RBOs were reintroduced solely within their historical range. Prior to embarking on a reintroduction program, the effect of anthropogenic threats on habitat distributions should be investigated as the habitat in the historical range may no longer be viable for current bird populations. Since 1988, 25 reintroductions (comprising a total of 1,359 birds) have occurred in South Africa. Postrelease monitoring is essential to determine the breeding status of RBOs at reintroduction sites and survival rates in human‐modified areas. Many landowners have switched to using oxpecker‐compatible tick control products and are keen to have these birds on their properties. The conservation status of RBOs has thus improved in South Africa. Their national conservation status has recently been changed to Least Concern, where they were previously listed as Near Threatened (Taylor, Peacock, & Wanless, 2015). Yet, RBO conservation remains a challenge at the wildlife/livestock interface outside of protected areas (Osofsky et al., 2005) and inside protected areas that are subject to high levels of poaching of large game (Ripple et al., 2015).

Many commercial and some rural subsistence farmers use dips, that is, acaracides at short intervals to keep their cattle virtually tick‐free. With the use of oxpecker‐compatible dips in conjunction with active reintroductions, the RBO has started to recover in some areas. With the help of the local farmers’ support, reintroductions will continue to restore RBO populations in areas within their historic range. Organophosphate‐based compounds should be avoided if communities wish to collectively increase RBO populations in their region. Conservation measures should make use of improved niche models that explicitly focus on abiotic–biotic factors, incorporating the complete ecology of the species, in predicting habitat suitability of other threatened mutualists (Dunn, Harris, Colwell, Koh, & Sodhi, 2009). To assess ecological planning for threatened species, it is important to account for the biotic interactions among multiple codependent species in niche models. This is especially true when multiple species are a part of the interactive network, and when predictions are reliable for conservation planning. Our results highlight areas within the country that are currently suitable for RBOs, with little or no interventions needed. However, in terms of possible sites for future reintroductions, areas in the Eastern Cape and southern KZN could be considered provided there is some woody cover and sufficient nest boxes are made available to the birds. Interfaces between protected and private land constitute sharp transitions between areas occupied by host communities that are extremely contrasted in terms of composition, diversity, and density. Our models also predicted habitat suitability outside of protected areas, suggesting that RBO conservation should focus on areas not formally protected. If private areas are well managed, with the cooperation of landowners regarding the use of oxpecker‐compatible agrochemicals, the long‐term survival of RBOs in South Africa seems promising. Our results provide a scientifically based platform for highlighting suitable habitat for birds, in our case, RBOs, to ensure their long‐term survival. We recommend that any reintroduction program first assesses the potential habitat currently available to the species in question, as our findings show that not all areas within a species’ historic range can be considered suitable for reintroductions.

## Conflict of Interest

None declared.

## Author Contributions

All authors developed the ideas for the manuscript together. CTD sourced funding. RK and LC sourced data. RK and TR did the analyses. RK drafted the manuscript, and TR, LC, and CTD made contributions.
