# Supplementary material for: Re‐establishing the pecking order: Niche models reliably predict suitable habitats for the reintroduction of red‐billed oxpeckers
Source: Ecol Evol. 2017 Feb 23;7(6):1974–83. doi: 10.1002/ece3.2787 (PMC5355191; doi:10.1002/ece3.2787)
Supplement: Supplementary file 1 [file ECE3-7-1974-s001.docx]

Appendix S1. A map showing presence/absence records of RBOs in South Africa, their historic range as per Stutterheim (1982) and their current IUCN range (BirdLife International 2012). Absence records shown were taken from SABAP 2.


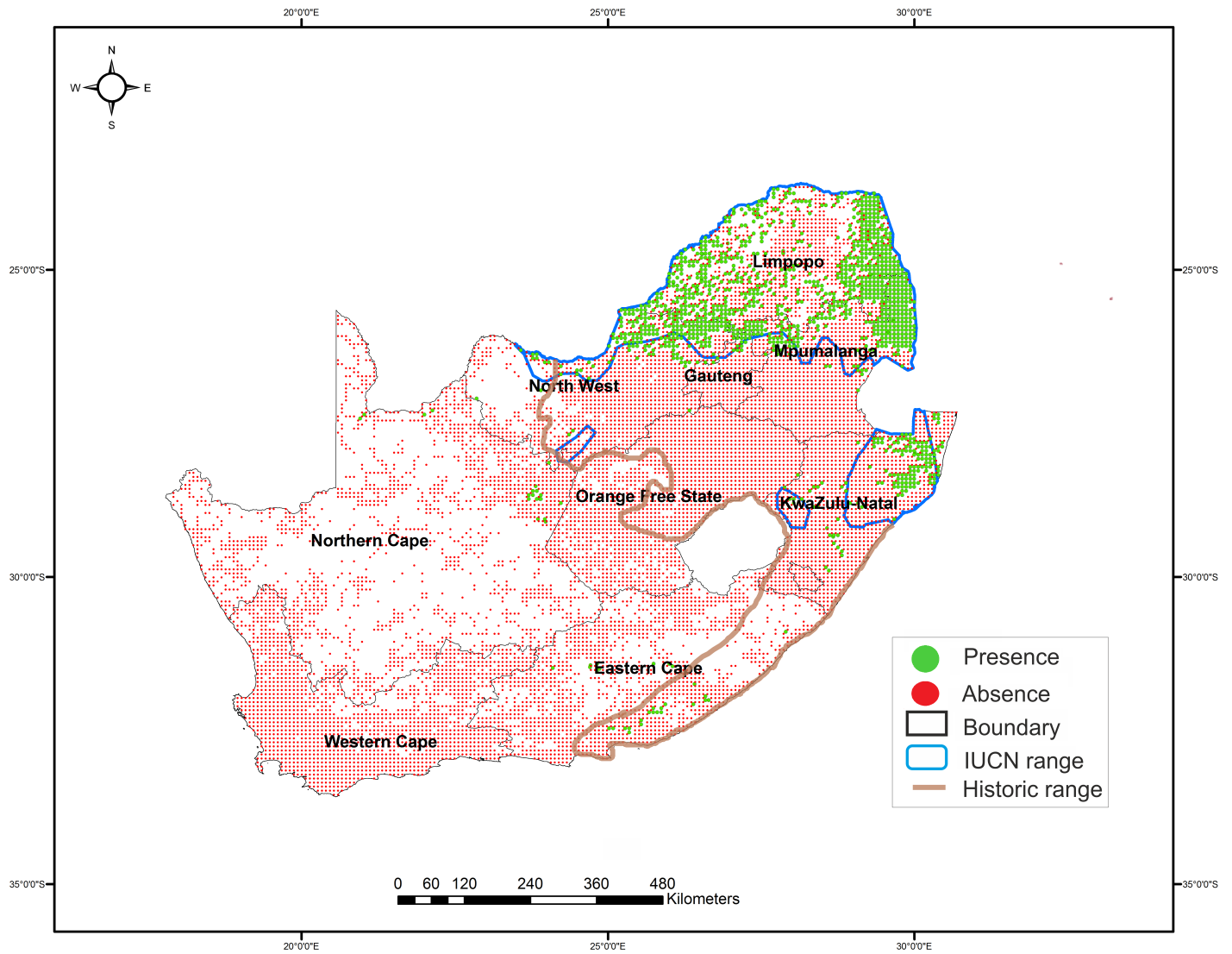


**References**

Stutterheim, C. J. 1982. Past and present ecological distribution of the redbilled oxpecker (*Buphagus erythrorhynchus*) in South Africa. S. Afr. J. Zool. 17:190–196.

BirdLife International. 2012. *Buphagus erythrorhynchus*. The IUCN Red List of Threatened Species 2012:e.T22711009A39697998.
