# Supplementary material for: Re‐establishing the pecking order: Niche models reliably predict suitable habitats for the reintroduction of red‐billed oxpeckers
Source: Ecol Evol. 2017 Feb 23;7(6):1974–83. doi: 10.1002/ece3.2787 (PMC5355191; doi:10.1002/ece3.2787)
Supplement: Supplementary file 2 [file ECE3-7-1974-s002.docx]

Appendix S2. Abiotic and biotic predictors considered in the distribution models for RBOs in South Africa.

**1. Biotic:** Wild host species density was calculated pooling the occurrence records of African buffalo *Syncerus caffer*, white rhino *Ceratotherium simum*, black rhino *Diceros bicornis*, giraffe *Giraffa camelopardalis*, plains zebra *Equus quagga*, mountain zebra *E. zebra*, eland *Tragelaphus oryx*, impala *Aepyceros melampus*, kudu *Tragelaphus strepsiceros*, warthog *Phacochoerus africanus*, nyala *Tragelaphus angasii*, bushbuck *T. scriptus*, sable *Hippotragus niger*, blue wildebeest *Connochaetes taurinus*, black wildebeest *C. gnou*, waterbuck *Kobus ellipsiprymnus*, roan antelope *Hippotragus equinus*, heartbeest *Alcelaphus buselaphus*, hippopotamus *Hippopotamus amphibius*, and African elephant *Loxodonta Africana*.

**Data source and references:** Occurrence records were obtained from the Ezemvelo KwaZulu-Natal Wildlife (<http://www.kznwildlife.com/database.html>), Mammal Map group (University of Cape Town) and Durban Natural Science Museum.

**Justification and/or methodology:** Mammal host species are useful perches for oxpeckers to feed on ticks, flies and other ectoparasites. The presence of a host animal is imperative for successful breeding (Plantan et al. 2014). Although many studies have reported preference for some host species, we did not limit our data to this as it has been reported that they have to rely on multiple host species (a variety of large and medium sized mammals) for food (Mooring and Mundy 1996; Koenig 1997). We browsed through Google Scholar literature that recorded any oxpecker interactions or associations with host species eg. feeding, scissoring, perching, pecking etc) based on which we considered 20 host species as potential wild symbionts (Stutterheim and Stutterheim 1980; Stutterheim 1981; Hart et al. 1990; Mooring and Mundy 1996; Koenig 1997; Weeks 1999; Plantan et al. 2014; Nunn et al. 2011). Host occurrence records were obtained from 2007 to 2014. All records were pooled and plotted in ArcMap 10 (ESRI 2012). Kernel density was calculated to represent an index of wild host density.

**Data type**: Continuous

**Code:** host density

**2.** **Biotic:** Starling density was calculated pooling records of black-bellied starling *Lamprotornis corruscus*, Cape glossy starling *L. nitens*, red-winged starling *Onychognathus morio*, violet-backed starling *Cinnyricinclus leucogaster*, wattled starling *Creatophora cinerea* and pied starling *Spreo* *bicolor*.

**Source/reference:** South African Bird Atlas Project 2 (<http://sabap2.adu.org.za/>)

**Justification and/or methodology:** RBO is a secondary cavity-nester, and relies on available cavities that occur naturally in the environment (Stutterheim 1976). Presence/absence of other starlings indicates potential nesting sites suitable for Sturnidae species which would be helpful in spatial predictions of RBO. Using associated species in distribution models also improves the predictive performance of the modelled/target species (Morelli and Tryjanowski 2015). Therefore, presence/absence records of other species of starling were obtained from SABAP2 and pooled the records together and plotted in ArcMap 10 (ESRI 2012). Kernel density was calculated to represent an index of starling density in South Africa.

**Data type:** Continuous

**3.** **Biotic:** Tick density was calculated pooling records of *Rhipicephalus* *appendiculatus*, *Amblyomma* *hebraeum*, *R.* (*B.*) *decoloratus*, *R.* (*B.*) *microplus*, *R.* *evertsi evertsi*, and *Hyalomma truncatum*.

**Source/reference:** Dr. Arthur Spickett provided recent tick occurrence data from 2007-2014 and in addition other records from the same time period were procured using the “spocc” package in R (Chamberlain et al. 2014).

**Justification and/or methodology**: Ticks form a major proportion in the diet of oxpeckers (Bezuidenhout and Stutterheim 1980), therefore variation in tick density could play a major role in the feeding ecology of RBO (Weeks 1999). Selected tick species were identified from literature wherein the aforementioned six tick species were preferred by RBO (Bezuidenhout and Stutterheim 1980; Weeks 2000; Plantan et al. 2014). We pooled all the tick records from 2007-2014 and calculated the Kernel density using the Spatial analyst tool in ArcMap 10 (ESRI 2012) to represent an index of tick density.

**Data type:** Continuous

**4.** **Climate:** Nineteen bioclimatic variables; bio 1 = annual mean temperature (°C), bio 2 = mean diurnal range (mean of monthly (max temp - min temp)), bio3 = isothermality (bio2/bio7) (* 100), bio4 = temperature seasonality (standard deviation *100), bio5 = maximum temperature of warmest month (°C), bio6 = minimum temperature of coldest month (°C), bio7 = temperature annual range (bio5-bio6), bio8 = mean temperature of wettest quarter (°C), bio9 = mean temperature of driest quarter (°C), bio10 = mean temperature of warmest quarter (°C), bio11 = mean temperature of coldest quarter (°C), bio12 = annual precipitation (mm), bio13 = precipitation of wettest month (mm), bio14 = precipitation of driest month (mm), bio15 = precipitation seasonality (coefficient of variation), bio16 = precipitation of wettest quarter (mm), bio17 = precipitation of driest quarter (mm), bio18 = precipitation of warmest quarter (mm), and bio19 = precipitation of coldest quarter (mm).

**Source/reference:** The global raster layers at 30 arc sec resolution for each bioclimatic layer was downloaded from http://www.worldclim.org/bioclim (Hijmans et al. 2005).

**Justification and/or methodology:** RBO occurs in areas with an annual rainfall > 500 mm (Stutterheim and Brooke 1981; Stutterheim 1982). Temperature and rainfall determine the distribution of ticks (Howell et al. 1978) and this could in turn drive distributions of oxpeckers. In Africa tick species require moist conditions for survival and reproduction (Londt and Whitehead 1972). Naturally oxpeckers tend to be found in slightly wetter areas in the savanna landscape. The global raster layers at 30 arc sec resolution for each bioclimatic layer was downloaded and then clipped to the extent of South Africa. The mean values for each bioclimatic variable was then quantified using Zonal statistics tool in ArcMap 10 (ESRI 2012).

**Data type**: Continuous

**5.** **Topography:** Aspect (East, West, North, South, Undefined). East: 45˚ < aspect ≤ 135˚, West: 225˚ < aspect ≤ 315˚, North: 0˚< aspect ≤45˚ or 315˚< aspect ≤360˚, South: 135˚ < aspect ≤ 225˚, Undefined: Slope aspect undefined; this value is used for grids where slope gradient is undefined or slope gradient is less than 2%.

**Source/reference:** The global raster layer at 30 arc sec resolution for each direction was downloaded from http://webarchive.iiasa.ac.at/Research/LUC/External-World-soil-database/HTML/global-terrain-slope-download.html?sb=7 (Fischer et al. 2008) and then clipped to the extent of South Africa.

**Justification and/or methodology:** Directionality can significantly affect the reproductive success of cavity nesting birds as it is likely related to the warm climate (Shwartz et al. 2009). The global raster layer at 30 arc sec resolution for each direction was downloaded and clipped to the extent of South Africa. The mean aspect for each category was then quantified using Zonal statistics tool in ArcMap 10 (ESRI 2012).

**Data type:** Continuous

**6.** **Topography**: Elevation (m)

**Source/Reference**: SRTM 90m Digital Elevation Database v4.1 (http://www.cgiar-csi.org/data/srtm-90m-digital-elevation-database-v4-1#download) from Jarvis et al. (2008).

**Justification and/or methodology**: RBO is found from 1400 to 2500 m in altitude along its range (Attwell 1966; Hustler 1987). Elevation is also one of the major limiting factors for tick distributions in Africa (Cumming 2002). The global SRTM 90m Digital Elevation model data v4.1 at 90 m resolution was downloaded and then clipped to the extent of South Africa. The mean elevation was then quantified using Zonal statistics tool in ArcMap 10 (ESRI 2012).

**7.** **Vegetation:** Land cover

**Source/Reference:** South African National Land Cover for 2014 obtained from http://bgis.sanbi.org/DEA_Landcover/project.asp (Geoterraimage 2015).

**Justification and/or methodology:** Although RBO has been recorded in 29 different vegetation types in South Africa (Acocks 1975) they favour the savanna and bushveld vegetation (Feare and Craig 1998). Species habitat preference moves along a gradient from open to closed habitat, thus mammalian hosts are exposed to increasing numbers of ticks along this gradient (Mooring et al. 2000). This in turn influences the rate at which ticks are consumed by RBO (Feare and Craig 1998). The original 72 categories were reclassified into 9 broad categories (water and wetlands, forest, thicket, woodland, grassland, fynbos, cultivation, plantation and urban).

**Data type**: Categorical

**8.** **Vegetation**: Biome

**Source/Reference:** National Spatial data was downloaded from http://bgis.sanbi.org/SpatialDataset/Detail/331 (South African National Biodiversity Institute 2012)

**Justification and/or methodology:** Hosts are more likely to be selected when in habitat types that increase the visibility of hosts to oxpeckers, such as in open grassland versus closed woodland (Mooring and Mundy 1996) and in terms of food availability. There were 11 categories originally, which were reclassified into nine categories (azonal vegetation, forests, water bodies, albany thicket, fynbos, savanna, nama-karoo, grassland, and coastal forest) for further modelling.

**Data type**: Categorical

**9.** **Vegetation**: Tree cover (%)

**Source/Reference:** The global raster layer Global Land Cover-SHARE of year 2014 - Beta-Release 1.0 was downloaded from http://www.glcn.org/databases/lc_glcshare_en.jsp as developed by Latham et al. (2014).

**Justification and/or methodology:** RBO’s preference for savanna is linked with the climatic conditions and the variety of host species (large mammals) found. The savannas are losing tree cover due to excessive browsing by large herbivores such as elephant and giraffe as well as fire incidents (Staver et al. 2009). Hence, adequate tree cover is essential for cavity nesting birds such as RBO that relies on tree holes for breeding therefore adequate tree cover is an important element in a suitable site. The global raster layer at 30 arc second resolution was downloaded and clipped to the extent of South Africa.

**Data type**: Continuous

**10.** **Water:** Distance to river (m)

**Source/Reference:** The polyline layer on rivers of Africa was derived from World Wildlife Fund's (WWF) HydroSHEDS (http://ref.data.fao.org/map?entryId=b891ca64-4cd4-4efd-a7ca-b386e98d52e8&tab=metadata)

**Justification and/or methodology:** Oxpeckers have been frequently sighted close to large rivers often where large game congregate (Stutterheim 1981). The national polyline feature was downloaded and then used to calculate Euclidean distance to river (km) using the Spatial Analyst Tool in ArcMap 10 (ESRI 2012).

**Data type:** Continuous

**11.** **Water:** Surface water body density

**Source/Reference:** Southern Africa Development Community (SADC)- WRD Surface Waterbodies database (Jenness et al. 2007a; Jenness et al. 2007b; http://ref.data.fao.org/map?entryId=eb0d2910-e362-11db-a939-000d939bc5d8&tab=metadata)

**Justification and/or methodology:** Surface water availability fluctuates seasonally causing a shift in local movements of large mammal symbionts and in areas where locally seasonal migration occurs and where water supply decreases we would expect oxpeckers to survive only by following the movement of their symbionts (Stutterheim 1981). Oxpeckers often perch on host species close to water bodies. They are often observed drinking from the same waterhole and simply fly down from the host to the water; and back again. Point feature data on lakes, dams and reservoir features were considered. Point data was merged and Kernel density was calculated in ArcMap 10 (ESRI 2012) to measure the surface water body density.

**Data type:** Continuous

**12.** **Proximity:** Distance to protected areas (m)

**Source/Reference:** UNEP-WCMC (2012). Data Standards for the World Database on Protected Areas. UNEP-WCMC: Cambridge, UK (http://www.protectedplanet.net/)

**Justification and/or methodology:** Oxpeckers are dependent on game species (hosts) which means that they are naturally common in protected parks and reserves. The world spatial data (polygon feature) was clipped to the extent of South Africa and Euclidean distance to protected areas (m) was calculated using the “Euclidean distance tool” in Spatial Analyst, ArcMap 10 (ESRI 2012).

**Data type:** Continuous

**References**

|  |
| --- |

Acocks, J. P. H. 1975. Veld types of South Africa. Memoirs of the Botanical Survey of South Africa., 40. Government Printer, Pretoria.

Attwell, R. I. G. 1966. Oxpeckers, and their associations with mammals in Zambia. Puku 4:117-148.

Bezuidenhout, J. D., and C. J. Stutterheim. 1980. A critical evaluation of the role played by the red-billed oxpecker *Buphagus erythrorhynchus* in the biological control of ticks. Onderstepoort J. Vet. Res. 47:51-75.

Chamberlain, S., K. Ram, and T. Hart. 2014. spocc: R interface to many species occurrence data sources. R package version 0.1.0. <https://github.com/ropensci/spocc>

Cumming, G. S. 2002. Comparing climate and vegetation as limiting factors for species ranges of African ticks. Ecology 83:255-268.

ESRI. 2012. ArcGIS Desktop: Release 10.0. Redlands, CA: Environmental Systems Research Institute.

Feare, C., and A. Craig. 1998. Starlings and mynas. Helm, London.

Fischer, G., F. O., Nachtergaele, S. Prieler, H. T., van Velthuizen, L. Verelst, and D. Wiberg. 2008. Global agro-ecological zones assessment for agriculture (GAEZ 2008) - IIASA, Laxenburg, Austria.

Geoterraimage. 2015. 2013 - 2014 South African National Land Data User Report and MetaData: a commercial data product created by Geoterraimage, version 05 Land-Cover Dataset. 53 pp

Hart, B. L., L. A. Hart, and M. S. Mooring. 1990. Differential foraging of oxpeckers on impala in comparison with sympatric antelope species. Afr. J. Ecol. 28:240–249.

Hijmans, R. J., S. E. Cameron, J. L. Parra, P. G. Jones, and A. Jarvis. 2005. Very high resolution interpolated climate surfaces for global land areas. Int. J. Climatol. 25:1965-1978.

Howell, C. J., J. B. Walker, and E. M. Nevill. 1978. Ticks, mites and insects infesting domestic animals in South Africa. Part 1. Descriptions and biology. Department of Agricultural Technical Services, Republic of South Africa. (Science Bulletin no. 393).

Hustler, K. 1987. Host preference of oxpeckers in the Hwange National Park Zimbabwe. Afr. J. Ecol. 25:241-245.

Jarvis, A., H. I. Reuter, A. Nelson, and E. Guevara. 2008. Hole-filled SRTM for the globe Version 4, available from the CGIAR-CSI SRTM 90m Database (http://srtm.csi.cgiar.org).

Jenness, J., J. Dooley, J. Aguilar-Manjarrez, and C. Riva. 2007a. African water resource database. GIS-based tools for inland aquatic resource management. 1. Concepts and application case studies CIFA Technical Paper. No.33, Part 1. Rome, FAO. 167p

Jenness, J., J. Dooley, J. Aguilar-Manjarrez, and C. Riva. 2007b. African water resource database. GIS-based tools for inland aquatic resource management. 2. Technical manual and workbook. CIFA Technical Paper. No. 33, Part 2. Rome, FAO. 308 p

Koenig, W. D. 1997. Host preferences and behaviour of oxpeckers: co-existence of similar species in a fragmented landscape. Evol. Ecol. 11:91-104.

Latham, J., R. Cumani, I., Rosati, and M. Bloise. 2014. Global land cover share (GLC-SHARE) database beta-release version 1.0-2014. FAO: Rome, Italy.

Londt, J. G. H., and G. B. Whitehead. 1972. Ecological studies of larval ticks in South Africa (Acarina: Ixodidae). Parasitology 65:469–490.

Mooring, M. S., and P. J. Mundy. 1996. Factors influencing host selection by yellow‐billed oxpeckers at Matobo National Park, Zimbabwe. Afr. J. Ecol. 34:177-188.

Mooring, M. S., J. E. Benjamin, C. R. Harte, and N. B. Herzog. 2000. Testing the interspecific body size principle in ungulates: the smaller they come, the harder they groom. Anim. Behav. 60:35-45.

Morelli, F., and P. Tryjanowski. 2015. No species is an island: testing the effects of biotic interactions on models of avian niche occupation. Ecol. Evol. 5:759-768.

Nunn, C. L., V. O. Ezenwa, C. Arnold, and W. D. Koenig. 2011. Mutualism or parasitism? Using a phylogenetic approach to characterize the oxpecker‐ungulate relationship. Evolution 65:1297-1304.

Plantan, T. B. 2009. Feeding behavior of wild and captive oxpeckers (*Buphagus spp*.): A case of conditional mutualism. PhD Thesis, University of Miami, Coral Gables, FL.

Plantan, T. B., M. J. Howitt, A. Kotzé, and M. S. Gaines. 2014. Breeding biology of red‐billed oxpeckers *Buphagus erythrorhynchus* at the National Zoological Gardens of South Africa. Int. Zoo. Yearb. 48:92-100.

Shwartz, A., D. Strubbe, C. J. Butler, E. Matthysen, and S. Kark. 2009. The effect of enemy-release and climate conditions on invasive birds: a regional test using the rose-ringed parakeet (*Psittacula krameri*) as a case study. Divers. Distrib. 15:310–318.

South African National Biodiversity Institute. 2012. Vegetation Map of South Africa, Lesotho and Swaziland 2009 update [vector geospatial dataset]. Available from the Biodiversity GIS website, downloaded on 26 August 2015

Staver, A. C., W. J. Bond, W. D. Stock, S. J. van Rensburg, and M. S. Waldram. 2009. Browsing and fire interact to suppress tree density in an African savanna. Ecol. Appl. 19:1909–1919.

Stutterheim, C. J. 1976. The biology of the red-billed oxpecker, *Buphagus erythrorhynchus* (Stanley 1814) in the Kruger National Park. MSc. Thesis, University of Pretoria.

Stutterheim, C. J. 1981. The movements of a population of redbilled oxpeckers (*Buphagus Erythrorhynchus*) in the Kruger National Park. Koedoe 24:99-107.

Stutterheim, C. J. 1982. Past and present ecological distribution of the redbilled oxpecker (*Buphagus erythrorhynchus*) in South Africa. S Afri. J. Zool. 17:190–196.

Stutterheim, C. J., and M. Stutterheim. 1980. Evidence of an increase in a red-billed oxpecker population in the Kruger National Park. S Afri. J. Zool. 15:284-284.

Stutterheim, C. J., and R. K. Brooke. 1981. Past and present ecological distribution of the redbilled oxpecker in South Africa. S Afri. J. Zool. 16:44-49.

UNEP-WCMC 2012. Data Standards for the World Database on Protected Areas. UNEP-WCMC: Cambridge, UK

Weeks, P. 1999. Interactions between red-billed oxpeckers, *Buphagus erythrorhynchus*, and domestic cattle, *Bos taurus*, in Zimbabwe. Anim. Behav. 58:1253-1259.

Weeks, P. 2000. Red-billed oxpeckers: vampires or tickbirds?. Behav. Ecol. 11:154-160.
