# Supplementary material for: Re‐establishing the pecking order: Niche models reliably predict suitable habitats for the reintroduction of red‐billed oxpeckers
Source: Ecol Evol. 2017 Feb 23;7(6):1974–83. doi: 10.1002/ece3.2787 (PMC5355191; doi:10.1002/ece3.2787)

Appendix S3. A correlation plot of predictor variables at occurrence points of RBOs in South Africa. Red ellipses indicate correlated predictors (≥ 0.7). Grey ellipses are uncorrelated variables.


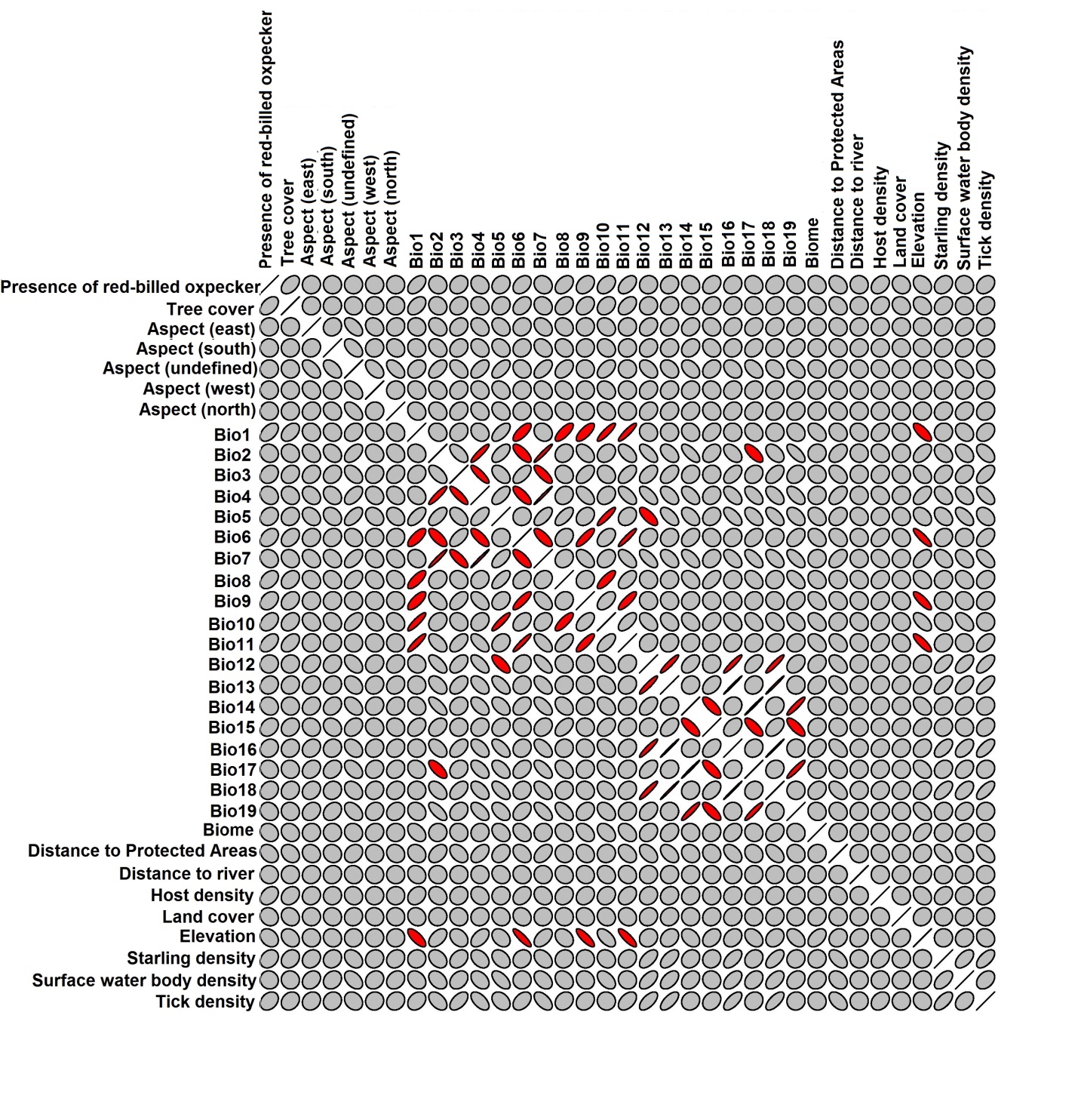

Supplement: Supplementary file 3 [file ECE3-7-1974-s003.docx]
