# Supplementary material for: Re‐establishing the pecking order: Niche models reliably predict suitable habitats for the reintroduction of red‐billed oxpeckers
Source: Ecol Evol. 2017 Feb 23;7(6):1974–83. doi: 10.1002/ece3.2787 (PMC5355191; doi:10.1002/ece3.2787)
Supplement: Supplementary file 4 [file ECE3-7-1974-s004.docx]

Appendix S4. Distribution models for RBOs in South Africa using GAM and GLM.

| **Category** | **GAM Model** | **df** | **logLik** | **AICc** | **ΔAICc** | **AICc**  **weight** |
| --- | --- | --- | --- | --- | --- | --- |
| abiotic + biotic (GAM_top) | aspect(west) + bio18 + bio7 + bio8 + bio9 + biome + distance to protected areas + elevation + host density + land cover + starling density + surface water body density + tick density + tree cover | 37.71 | -1469.65 | 3015.08 | 0.00 | 0.79 |
| abiotic + biotic | aspect(south) + bio18 + bio7 + bio8 + bio9 + biome + distance to protected areas + elevation + host density + land cover + starling density + surface water body density + tick density + tree cover | 38.49 | -1470.19 | 3017.75 | 2.67 | 0.21 |
| abiotic + biotic | bio18 + bio7 + bio8 + bio9 + biome + distance to protected areas + elevation + host density + land cover + starling density + surface water body density + tick density | 36.21 | -1479.89 | 3032.54 | 17.46 | 0.00 |
| abiotic + biotic | aspect(south) + bio18 + bio8 + bio9 + biome + distance to protected areas + elevation + host density + land cover + starling density + surface body density + tick density + tree cover | 38.87 | -1480.52 | 3039.16 | 24.08 | 0.00 |
| abiotic + biotic | bio18 + bio7 + bio8 + bio9 + biome + elevation + host density + land cover + starling density + surface water body density + tick density + tree cover | 34.29 | -1501.58 | 3072.05 | 56.97 | 0.00 |
| abiotic + biotic | bio18 + bio7 + bio8 + bio9 + biome + elevation + host density + starling density + surface water body density + tick density + tree cover | 26.78 | -1532.88 | 3119.51 | 104.43 | 0.00 |
| abiotic + biotic | bio18 + bio7 + bio8 + bio9 + elevation + host density + starling density + surface water body density + tick density + tree cover | 29.60 | -1541.92 | 3143.27 | 128.19 | 0.00 |
| abiotic + biotic | bio18 + bio19 + bio7 + bio8 + bio9 + host density + starling density + tick density + tree cover | 26.80 | -1551.51 | 3156.80 | 141.72 | 0.00 |
| abiotic + biotic | bio18 + bio7 + bio8 + bio9 + elevation + host density + starling density + tick density + tree cover | 27.81 | -1553.28 | 3162.40 | 147.32 | 0.00 |
| biotic + climate | bio17 + bio18 + bio19 + bio7 + bio8 + bio9 + host density + starling density + tick density | 27.25 | -1570.52 | 3195.75 | 180.66 | 0.00 |
| climate | bio18 + bio19 + bio7 + bio8 + bio9 | 17.45 | -1713.63 | 3462.23 | 447.15 | 0.00 |
| climate | bio17 + bio18 + bio19 + bio7 + bio8 + bio9 | 15.58 | -1722.53 | 3476.29 | 461.20 | 0.00 |
| vegetation | biome + land cover + tree cover | 10.68 | -1966.34 | 3954.07 | 938.99 | 0.00 |
| vegetation + water | biome + land cover + distance to river + surface water body density | 13.46 | -2099.42 | 4225.81 | 1210.72 | 0.00 |
| topography | aspect(east) + aspect(south) + aspect(west) + aspect(north) + elevation | 10.00 | -2672.36 | 5364.75 | 2349.66 | 0.00 |
| proximity | distance to protected areas + distance to river | 5.21 | -2357.65 | 4725.73 | 1710.65 | 0.00 |
| biotic | host density + starling density + tick density | 11.50 | -2337.91 | 4698.86 | 1683.78 | 0.00 |
| topography + water | aspect(east) + aspect(south) + aspect(west) + aspect(north) + elevation + distance to river + surface water body density | 14.00 | -2618.36 | 5264.77 | 2249.69 | 0.00 |
| **Category** | **GLM Model** | **df** | **logLik** | **AICc** | **ΔAICc** | **AICc**  **weight** |
| abiotic + biotic (GLM_top) | bio17 + bio18 + bio7 + bio8 + bio9 + biome + distance to protected areas + elevation + host density + land cover + starling density + surface water body density + tick density | 28 | -1596.76 | 3249.72 | 0 | 0.76 |
| abiotic + biotic | aspect (west) + bio18 + bio7 + bio8 + bio9 + biome + distance to protected areas + elevation + host density + land cover + starling density + surface water body density + tick density + tree cover | 29 | -1597.09 | 3252.41 | 2.69 | 0.20 |
| abiotic + biotic | aspect south + bio18 + bio7 + bio8 + bio9 + biome + distance to protected areas + elevation + host density + land cover + starling density + surface water body density + tick density + tree cover | 29 | -1598.57 | 3255.36 | 5.64 | 0.05 |
| abiotic + biotic | bio18 + bio7 + bio8 + bio9 + biome + distance to protected areas + elevation + host density + land cover + starling density + surface water body density + tick density | 27 | -1611.78 | 3277.75 | 28.03 | 0 |
| abiotic + biotic | bio18 + bio7 + bio8 + bio9 + biome + elevation + host density + land cover + starling density + surface water body density + tick density + tree cover | 27 | -1627.85 | 3309.89 | 60.17 | 0 |
| abiotic + biotic | bio18 + bio7 + bio8 + bio9 + biome + elevation + host density + starling density + surface water body density + tick density + tree cover | 19 | -1666.86 | 3371.82 | 122.1 | 0 |
| abiotic + biotic | bio18 + bio19 + bio7 + bio8 + bio9 + host density + starling density + tick density + tree cover | 10 | -1697.02 | 3414.07 | 164.35 | 0 |
| abiotic + biotic | bio18 + bio7 + bio8 + bio9 + elevation + host density + starling density + surface water body density + tick density + tree cover | 11 | -1706.53 | 3435.1 | 185.38 | 0 |
| abiotic + biotic | bio18 + bio7 + bio8 + bio9 + elevation + host density + starling density + tick density + tree cover | 10 | -1707.75 | 3435.53 | 185.81 | 0 |
| climate + biotic | bio17 + bio18 + bio19 + bio7 + bio8 + bio9 + host density + starling density + tick density | 10 | -1712.55 | 3445.12 | 195.4 | 0 |
| climate | bio17 + bio18 + bio7 + bio8 + bio9 | 6 | -1910.38 | 3832.78 | 583.06 | 0 |
| climate | bio18 + bio19 + bio7 + bio8 + bio9 | 6 | -1923.34 | 3858.69 | 608.98 | 0 |
| vegetation | land cover + biome + tree cover | 18 | -1918.59 | 3873.26 | 623.54 | 0 |
| water + vegetation | biome + land cover + distance to water + surface water body density | 19 | -2042.85 | 4123.79 | 874.07 | 0 |
| topography | aspect (east) + aspect (south) + aspect (west) + aspect (north) + elevation | 6 | -2754.01 | 5520.04 | 2270.32 | 0 |
| proximity | distance to protected areas + distance to river | 3 | -2468.67 | 4943.35 | 1693.63 | 0 |
| biotic | host density + starling density + tick density | 4 | -2569.98 | 5147.96 | 1898.24 | 0 |
| topography + water | aspect (east) + aspect (south) + aspect (west) + aspect (north) + elevation + distance to river + surface water body density | 8 | -2750.18 | 5516.37 | 2266.65 | 0 |

Parameters included are the model’s degrees of freedom (df) and Loglikelihood value (logLik). For explanation of variable codes refer to Appendix S2
