# Supplementary material for: Re‐establishing the pecking order: Niche models reliably predict suitable habitats for the reintroduction of red‐billed oxpeckers
Source: Ecol Evol. 2017 Feb 23;7(6):1974–83. doi: 10.1002/ece3.2787 (PMC5355191; doi:10.1002/ece3.2787)
Supplement: Supplementary file 5 [file ECE3-7-1974-s005.docx]

Appendix S5. Figures showing the response plots from GAM (top model), GLM (top model) and BRT and a table showing the relative contribution of variables for predicting the distribution of RBO.

Figure S1. Smoothed partial dependence plots for the top GAM. For each plot, there is a greater chance of occurrence of RBOs than absence, where y > 0 and a greater chance of absence than presence when y < 0, holding all other variables at their mean values.


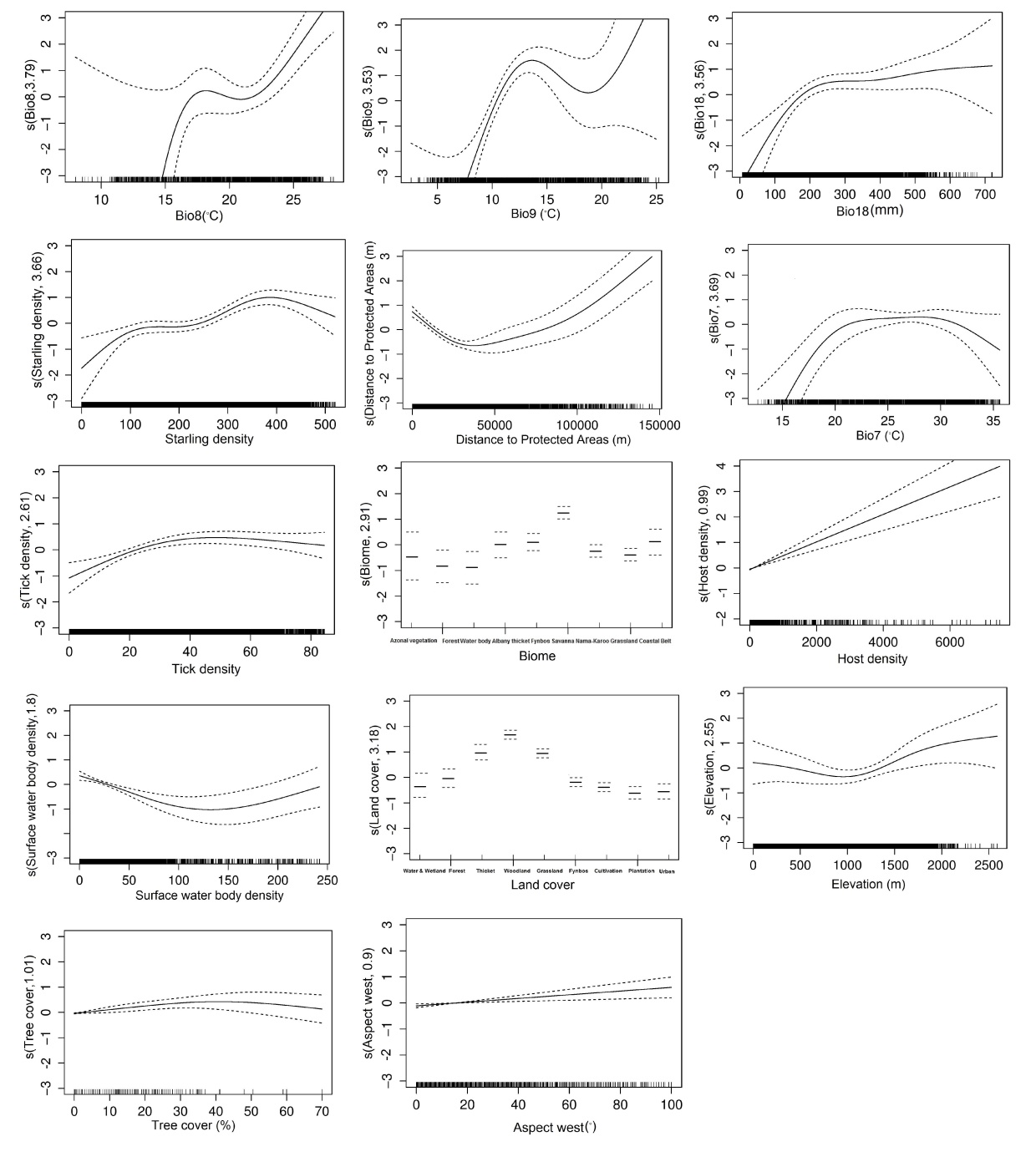


Figure S2. Effects plots for the top GLM showing predicted probabilities of RBO occurrence against predictors in the top model.


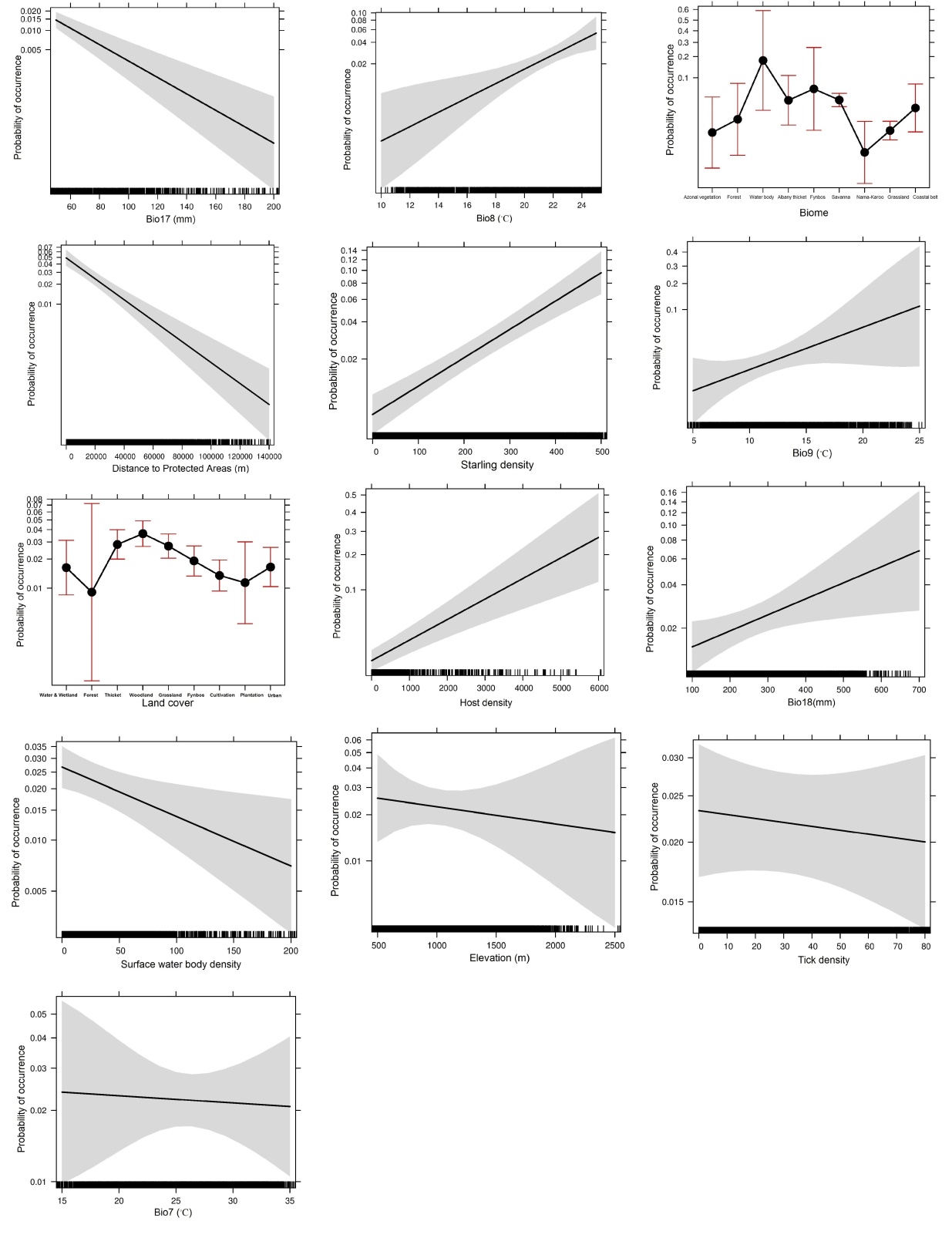


Figure S3. Partial response plots for the most influential variables in BRT of RBOs. Y-axes are changes in the log odds of presence of RBOs and are centered to have zero mean over the data distribution. For each plot, there is a greater chance of species presence than absence where y > 0 and a greater chance of species absence than presence when y < 0, holding all other variables at their mean values.


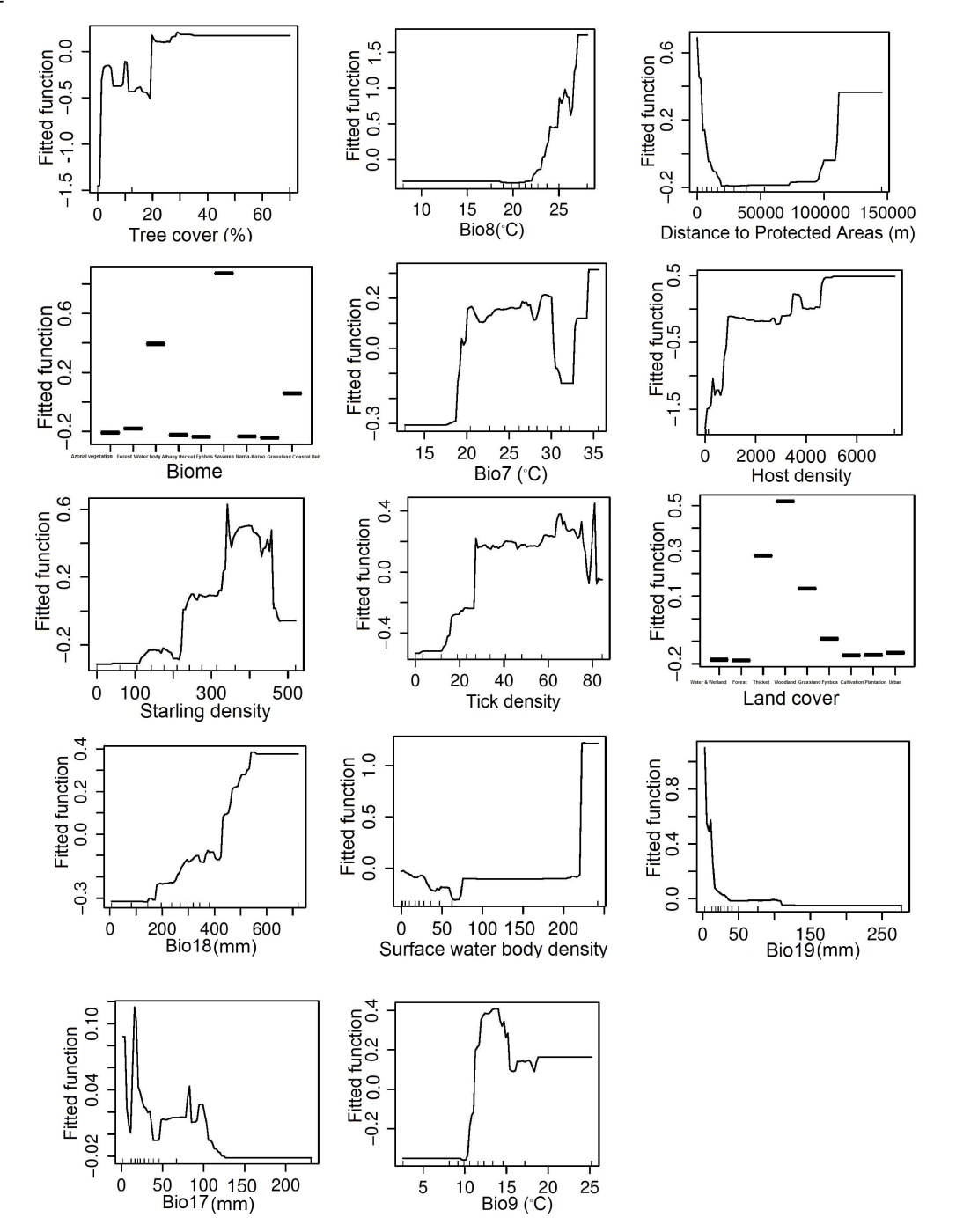


Table S1. The relative importance (%) of variables in the distribution models (BRT, GAM and GLM) of RBO in South Africa.

| **Variable** | **BRT** | **GAM** | **GLM** |
| --- | --- | --- | --- |
| Tree cover | 25.9 | 0.6 | - |
| Bio8 | 11.5 | 30 | 16 |
| Distance to Protected Areas | 10.2 | 6.1 | 14.6 |
| Biome | 8.8 | 3.5 | 15.2 |
| Bio7 | 7.4 | 4.4 | 0.03 |
| Host density | 6.5 | 3.5 | 3.9 |
| Starling density | 5.9 | 7.5 | 13.4 |
| Tick density | 5.2 | 3.9 | 0.04 |
| Land cover | 4.2 | 2.1 | 5.2 |
| Bio18 | 3.5 | 9.5 | 2.4 |
| Surface water body density | 2.8 | 2.3 | 1.35 |
| Bio19 | 2.7 | - | - |
| Bio17 | 2.5 | - | 19.7 |
| Bio9 | 2.5 | 25 | 7.2 |
| Elevation | - | 1.4 | 0.44 |
| Aspect(west) | - | 0.3 | - |

- = variables absent in the model
