# Supplementary material for: Re‐establishing the pecking order: Niche models reliably predict suitable habitats for the reintroduction of red‐billed oxpeckers
Source: Ecol Evol. 2017 Feb 23;7(6):1974–83. doi: 10.1002/ece3.2787 (PMC5355191; doi:10.1002/ece3.2787)
Supplement: Supplementary file 6 [file ECE3-7-1974-s006.docx]

Appendix S6. Figure and table shows the performance and evaluation of species distribution models for RBO

Figure S4. Calibration plots from ensemble models, boosted regression tree (BRT), generalized additive model (GAM), and generalized linear model (GLM) fitted for predicting RBO distribution. Points falling along the diagonal line indicate that models predicted the probability of RBO occurrence adequately.


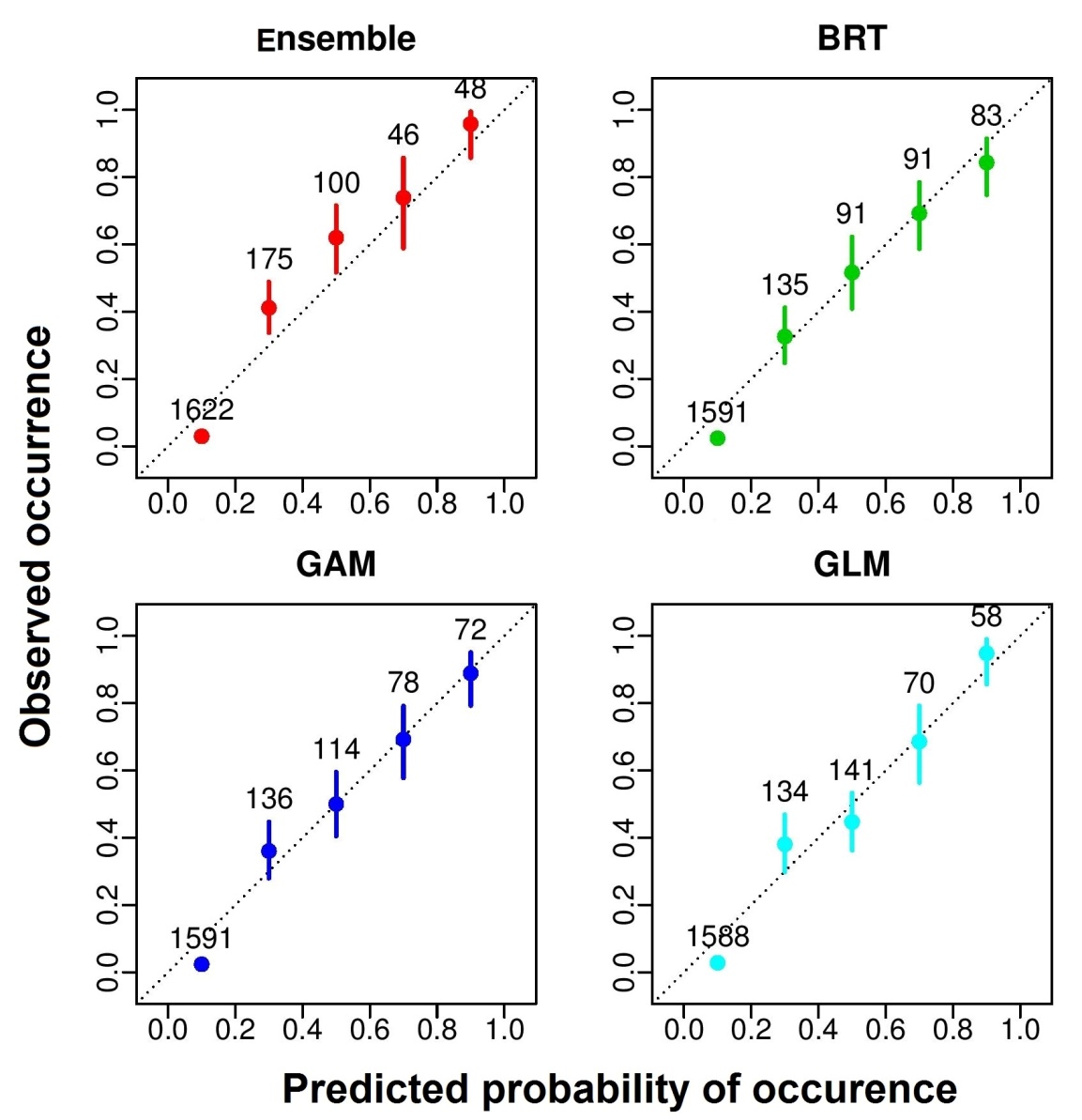


Table S2. Evaluation metrics for the best models from ensemble models, boosted regression tree (BRT), generalized additive model (GAM), and generalized linear model (GLM). PCC is the proportion of presences and absences correctly classified; sensitivity is the proportion of presences correctly classified; specificity is the proportion of absences correctly classified; AUC is the area under the curve.

| **Model** | **Threshold** | **PCC** | **Sensitivity** | **Specificity** | **Kappa** | **AUC** | **PCC.sd** | **Sensitivity.sd** | **Specificity.sd** | **Kappa.sd** | **AUC.sd** |
| --- | --- | --- | --- | --- | --- | --- | --- | --- | --- | --- | --- |
| Ensemble | 0.5 | 0.91 | 0.41 | 0.98 | 0.50 | 0.94 | 0.0065 | 0.03 | 0.0031 | 0.032 | 0.0067 |
| BRT | 0.5 | 0.92 | 0.59 | 0.97 | 0.60 | 0.94 | 0.0062 | 0.03 | 0.0044 | 0.028 | 0.0067 |
| GAM | 0.5 | 0.91 | 0.56 | 0.97 | 0.58 | 0.94 | 0.0063 | 0.031 | 0.0043 | 0.029 | 0.0067 |
| GLM | 0.5 | 0.91 | 0.52 | 0.97 | 0.55 | 0.93 | 0.0065 | 0.031 | 0.0042 | 0.03 | 0.0075 |
